# The World Health Organization Intersectoral Global Action Plan on Epilepsy and Other Neurological Disorders and the headache revolution: from headache burden to a global action plan for headache disorders

**DOI:** 10.1186/s10194-023-01700-3

**Published:** 2024-01-04

**Authors:** Matilde Leonardi, Paolo Martelletti, Rami Burstein, Arianna Fornari, Licia Grazzi, Alla Guekht, Richard B. Lipton, Dimos Dimitrios Mitsikostas, Jes Olesen, Mayowa Ojo Owolabi, Elena Ruiz De la Torre, Simona Sacco, Timothy J. Steiner, Nirmal Surya, Takao Takeshima, Cristina Tassorelli, Shuu-Jiun Wang, Tissa Wijeratne, Shengyuan Yu, Alberto Raggi

**Affiliations:** 1 Neurology, Public Health and Disability Unit, Fondazione IRCCS Istituto Neurologico Carlo Besta, Via Celoria 11, 20133 Milan, Italy; 2 Unitelma Sapienza University of Rome, Rome, Italy; 3 John Hedley-Whyte Professor of Anesthesia and Neuroscience at the Beth Israel Deaconess Medical Center and Harvard Medical School, Boston, MA USA; 4 Neuroalgology Unit and Headache Center, Fondazione IRCCS Istituto Neurologico Carlo Besta, Milan, Italy; 5 Moscow Research and Clinical Center for Neuropsychiatry, Pirogov Russian National Research Medical University, Moscow, Russia; 6 Montefiore Headache Center and the Albert Einstein College of Medicine, New York, Bronx USA; 7 1st Neurology Department, Eginition Hospital, Medical School, National and Kapodistrian University of Athens, Athens, Greece; 8 Department of Neurology, Danish Headache Center, Rigshospitalet Glostrup, Faculty of Health and Medical Sciences, University of Copenhagen, Copenhagen, Denmark; 9 Faculty of Clinical Sciences, Center for Genomic and Precision Medicine, College of Medicine, University of Ibadan, Ibadan, Nigeria; 10 European Migraine and Headache Alliance, Brussels, Belgium; 11 Department of Biotechnological and Applied Clinical Sciences, University of L‘Aquila, L‘Aquila, Italy; 12 Department of Neuromedicine and Movement Science, Norwegian University of Science and Technology, Edvard Griegs gate, Trondheim, Norway; 13 Department of Neurology, Faculty of Health and Medical Sciences, University of Copenhagen, Glostrup, Denmark; 14 Division of Brain Sciences, Imperial College London, London, UK; 15 Surya Neuro Centre Mumbai, Mumbai, India; 16 Department of Neurology, Headache Center, Tominaga Hospital, Osaka, Japan; 17 Department of Brain and Behavioral Sciences, University of Pavia, Pavia, Italy; 18 Headache Science and Neurorehabilitation Center, IRCCS Mondino Foundation, Pavia, Italy; 19 College of Medicine and Brain Research Center, National Yang Ming Chiao Tung University, Taipei, Taiwan; 20 Department of Neurology, The Neurological Institute, Taipei Veterans General Hospital, Taipei, Taiwan; 21 Department of Neurology, Sunshine Hospital, St Albans, VIC Australia; 22 Australian Institute of Migraine, Pascoe Vale South, Victoria, Australia; 23 Department of Neurology, Chinese PLA General Hospital, Beijing, China

**Keywords:** Migraine, Tension-type headache, Global burden of disease study, World Health Organization, Brain health, Health promotion, Global campaign against headache

## Abstract

The World Health Organization (WHO) Intersectoral Global Action Plan on Epilepsy and Other Neurological Disorders was developed by WHO to address the worldwide challenges and gaps in provision of care and services for people with epilepsy and other neurological disorders and to ensure a comprehensive, coordinated response across sectors to the burden of neurologic diseases and to promote brain health across life-course. Headache disorders constitute the second most burdensome of all neurological diseases after stroke, but the first if young and midlife adults are taken into account. Despite the availability of a range of treatments, disability associated with headache disorders, and with migraine, remains very high. In addition, there are inequalities between high-income and low and middle income countries in access to medical care. In line with several brain health initiatives following the WHOiGAP resolution, herein we tailor the main pillars of the action plan to headache disorders: (1) raising policy prioritization and strengthen governance; (2) providing effective, timely and responsive diagnosis, treatment and care; (3) implementing strategies for promotion and prevention; (4) fostering research and innovation and strengthen information systems. Specific targets for future policy actions are proposed. The Global Action Plan triggered a revolution in neurology, not only by increasing public awareness of brain disorders and brain health but also by boosting the number of neurologists in training, raising research funding and making neurology a public health priority for policy makers. Reducing the burden of headache disorders will not only improve the quality of life and wellbeing of people with headache but also reduce the burden of neurological disorders increasing global brain health and, thus, global population health.

## Background

The World Health Organization Intersectoral Global Action Plan on Epilepsy and Other Neurological Disorders 2022–2031 (WHOiGAP) was developed by the World Health Organization (WHO) in consultation with Member States and other key stakeholders, including people living with neurological disorders, and endorsed by the 75th World Health Assembly in May 2022 under decision WHA 75 [1]. The action plan addresses the challenges and gaps in providing care and services for people with epilepsy and other neurological disorders that exist worldwide and seeks to promote a comprehensive, coordinated response across sectors. It includes actions to be undertaken by all stakeholders to attain global strategic objectives that address issues such as policy and governance; effective, timely and responsive diagnosis, treatment and care; promotion and prevention; research, innovation and information systems; and a public health response to epilepsy and other neurological disorders. The indicators for assessing progress towards meeting these global targets call upon Member States to monitor their policies and programmes for neurological disorders, and they set out reporting needs regarding a specific subset of information. As targets are voluntary and global, Member States are not necessarily expected to achieve all the specific targets individually but can contribute to a varying extent towards reaching them jointly. The global targets established for each strategic objective provide the basis for measurable collective action and progress by Member States towards global goals.

After one year, what progresses there have been regarding headache disorders? The headache global community has been participating in this global effort and the collaboration lies in years of common work between WHO and the scientific and lay communities of headache-related stakeholders. It was the beginning of the new century when, following the introduction of migraine and tension-type headache (TTH) into the Global Burden of Diseases (GDB) studies in 2001, that the Global Campaign against Headache “Lifting the Burden” (LTB) was launched – its purpose being to bring better health care to people with headache, thereby reducing the burden of headache worldwide [2, 3]. The pillars supporting this effort, which jointly engaged international organizations, academic institutions, patients’ organizations and other stakeholders, are that headache disorders are not only highly prevalent and around the world, but also that they are to a large extent treatable (https://www.l-t-b.org/go/the_global_campaign.background_history.the_burden_of_headache.html). Three main objectives were implemented by LTB as well as by WHO and other stakeholders through the years: collecting and collating evidence on the scope and scale of headaches-attributed burden, promoting awareness on these and, finally, developing evidence-based recommendations for interventions.

After 20 years of international efforts, and many publications, it is now well established that the burden of headache disorders, and of migraine in particular, is huge and multifaceted [4] as consequence of their prevalence and impacts on health and daily life. Of the two main primary headaches, TTH and migraine, TTH is much more common: in 2019 its age-standardized prevalence was 25,113/100,000 (95% UI: 22,021 to 28,316), whereas that of migraine was 14,107 (95% UI: 12,270 to 16,239) (see: https://vizhub.healthdata.org/gbd-results?params=gbd-api-2019-permalink/f603af7c89b9d2d2fd4b3d6a5066263f). Between 1990 and 2019, these estimates had increased by 0.3% (95% UI: -0.5 to 1.1%) for TTH and by 2.8% (95% UI: 1.8–3.9%) for migraine (see: https://vizhub.healthdata.org/gbd-results?params=gbd-api-2019-permalink/22e10043ea9212a7b0f9eeb265dcea3e) [5, 6], which, paradoxically, was likely an effect of headache awareness raising made by the Global Campaign against Headache [2]. How has this translated into the lived experience of having headache, i.e. in terms of years lived with disability (YLDs)? YLD rates attributed in 2019 to TTH were far fewer than those attributed to migraine: the former was 56.2/100,000 (95% UI: 17.0 to188.5), the latter was 525.5 (95% UI: 78.8 to 1,194.0) (see: https://vizhub.healthdata.org/gbd-results?params=gbd-api-2019-permalink/c4bb4e6f6c2f927894e896d3b545108c). YLD rates were basically stable over the 1990–2019 period, being the variation 0.5%, (95% UI -2.7 to 4.3%) for TTH, 4.6%, (95% UI -1.2 to 6.4%) for migraine (see: https://vizhub.healthdata.org/gbd-results?params=gbd-api-2019-permalink/9c18b0964a7189c550cb4b17013968d0) [5, 6]. However, over the last 30 years, available treatments for headache disorders, and for migraine in particular, have dramatically increased with a wide range of acute and prophylactic treatments, both pharmacological and non-pharmacological [7–13]. For almost all people affected by headache disorders, effective treatments exist. So, why is the burden still so high? The answer to this question appears to lie mostly in failures in health policy. As the Global Campaign states in its background “Not so much is known about the public-health aspects of headache disorders in poorer countries. The huge financial costs of headache focus attention on the developed world where money is persuasive. Costs to society of lost work-time may be less where labour costs are lower, but the burdens on people who are unable to work or care for their children can still be severe. No one should think that the humanitarian burdens of headache – not only pain, suffering and disability but also the many secondary burdens of lifestyle compromises, damaged relationships, and lost opportunities – weigh less elsewhere because they are less evident”.

Given these premises, how will WHOiGAP impact on headache policies worldwide and how can the headache community support the implementation of WHOiGAP at global level? To address this complex question, the co-authors of this paper, long-time headache clinicians, scientists and advocates for people living with headache, provide a comprehensive analysis of approaches to the objectives of the WHOiGAP as applied to headache disorders. The authors of this paper have contributed to the increase knowledge of headache as public health problem in different and complementary ways, either by leading specific initiatives, or by holding key position in scientific societies, by implementing data in the global burden study, or by leading patients’ organizations that worked on the WHOiGAP; some of the authors had an active and direct role in drafting the WHOiGAP or in co-authoring part of the material on which the WHOiGAP has been developed; all of them have been contributing to make headache a public health issue that deserves a global action plan.

## Moving from evidence on burden to public health actions on systems

The global failure to reduce the burden of headache disorders is largely due to a failure in make effective and cost-effective treatments available to all of those who need them. In most countries, this represents inadequate processes of care delivery, including proper diagnosis and appropriate allocation of treatment. The poor organization of headache services and its inequality around the globe [14–16] requires a rethinking of headache care as a whole [17]. Because this problem is common across many neurological disorders, the solution may require a wider approach to brain health [18].

WHOiGAP is intended to reduce the stigma, impact and burden of neurological disorders, including their associated mortality, morbidity and disability, and to improve the quality of life of people with neurological disorders, their caregivers and families, through a global approach to neurological disorders [1]. This, in turn, should also lead to an improvement of global brain health [18]. The broad term “neurological disorders” in WHOiGAP denotes conditions of the central and peripheral nervous systems and includes epilepsy, cerebrovascular diseases, headache disorders, neurodegenerative, neuroinfectious, neuroimmunological, neuromuscular, and neurodevelopmental disorders, spinal cord injury and traumatic brain injury (TBI), and cancers of the nervous system. WHOiGAP assumes a holistic approach to account for medical, individual, social and environmental influences; i.e. functioning and disability are considered the result of interactions between neurological conditions and contextual factors across the life course (as defined by the biopsychosocial model of health and disability of the WHO International Classification of Functioning, Disability and Health [19]) and burden is the result at the societal level of the amount of disability and mortality associated with a condition. Thus, to reduce the burden attributable to neurological disorders, WHOiGAP proposes an integrated, person-centred framework for the prevention, diagnosis, treatment, and care of people with neurological disorders, rather than a disease-specific approach.

The vision of WHOiGAP is declined into three pillars: (a) brain health is valued, promoted, and protected across the life course; (b) neurological disorders are prevented, diagnosed and treated, and premature mortality and morbidity are avoided; (c) people affected by neurological disorders and their carers attain the highest possible level of health, with equal rights, opportunities, respect and autonomy. For all neurological disorders, including headaches, four strategic objectives (the fifth is specific only for epilepsy) are identified as the route to this vision, and the overall goal of reducing the stigma, impact and burden of neurological disorders:

1. raising policy prioritization and strengthening governance;
2. providing effective, timely and responsive diagnosis, treatment and care;
3. implementing strategies for promotion and prevention;
4. fostering research and innovation and strengthening information systems;

Each of these objectives is to be achieved through a set of specific actions and global targets with indicators that guide the progress of activities and their success. Table 1 presents a synopsis of WHOiGAP objectives, actions and targets.

**Table 1:** A synopsis on WHOiGAP strategic objectives, actions areas and global targets

Strategic objectives & Actions Areas	Global Targets	
1) Raising policy prioritization and strengthen governance;	1.1) 75% of countries will have adapted or updated existing national policies, strategies, plans or frameworks to include neurological disorders by 2031.	1.2) 100% of countries will have at least one functioning awareness campaign or advocacy programme for neurological disorders by 2031.
1.1) Advocacy		
1.2) Policy, Plans and Legislation		
1.3) Financing		
2) Providing effective, timely and responsive diagnosis, treatment and care;	2.1) 75% of countries will have included neurological disorders in the UHC benefits package by 2031.	2.2) 80% of countries will provide the essential medicines and basic technologies required to manage neurological disorders in primary care by 2031.
2.1) Care pathways		
2.2) Medicines, diagnostics and other health products		
2.3) Health workers’ capacity-building, training and support		
2.4) Carer support		
3) Implementing strategies for promotion and prevention;	3.1) 80% of countries will have at least one functioning intersectoral programme for brain health promotion and the prevention of neurological disorders across the life course by 2031.	3.2) The global targets relevant for prevention of neurological disorders are achieved, as defined in: – the NCD-GAP; – Defeating meningitis by 2030: a global road map; and – Every newborn: an action plan to end preventable deaths.
3.1) Promoting healthy behaviour across the life course		
3.2) Infectious disease control		
3.3) Preventing head/spinal trauma and associated disabilities		
3.4) Reducing environmental risks		
3.5) Promotion of optimal brain development in children and adolescents		
4) Fostering research and innovation and strengthen information systems;	4.1) 80% of countries routinely collect and report on a core set of indicators for neurological disorders through their national health data and information systems at least every three years by 2031.	4.2) The output of global research on neurological disorders doubles by 2031.
4.1) Investment in research		
4.2) Data and information systems		
5) Strengthening the public health approach to epilepsy.	5.1) By 2031, countries will have increased service coverage for epilepsy by 50% from the current coverage in 2021.	5.2) 80% of countries will have developed or updated their legislation with a view to promoting and protecting the human rights of people with epilepsy by 2031.
5.1) Access to services for epilepsy		
5.2) Engagement and support for people with epilepsy		
5.3) Epilepsy as an entry point for other neurological disorders		

All Strategic objectives, Actions Areas and Global Targets are valid for neurological disorders as a whole, with the exclusion of the last one which is tailored on epilepsy only, and was therefore not further on discussed in the present manuscript. *UHC *Universal Health Coverage, *NCD-GAP *Non-Communicable Disorders Global Action Plan

## WHOiGAP strategic objectives and their impact on global headache strategies worldwide

The implementation of WHOiGAP relies on actions that both Member States and relevant stakeholders are expected to undertake to reach the strategic objectives in the next years. To reduce the burden of headache disorders and thereby improve global brain health among populations, it is necessary to link the global targets and indicators, useful to reach each strategic objective, to a set of elements, including: population measures of prevalence and burden, to health service availability, to resources, and, in light of these, to strategies that Member States can reasonably adopt. The indicators provided by the WHO are a useful pathway that can be adapted to headache disorders and implemented worldwide in a global action plan against this heterogeneous group of conditions.

Much has been done to address the targets of WHOiGAP through several actions, documents, initiatives, but even more has still to be done to improve health, quality of life and wellbeing of people with headache worldwide. The objectives and targets here presented should not be considered as indications for direct actions: rather they constitute general public health and policy guidelines.

### Strategic objective 1: raising policy prioritization and strengthen governance

Enhancing the priority of headache disorders within political agendas is expected to have great impact on population health, since they directly affect 35% (95% UI: 32–38%) of globally (see: https://vizhub.healthdata.org/gbd-results?params=gbd-api-2019-permalink/1fa43dac68a7d8f3b9e399f281fe84a7), a percentage that rises to 50% (95% UI: 43–57%) among females in the age group 35–39 years (see: https://vizhub.healthdata.org/gbd-results?params=gbd-api-2019-permalink/66450ec93908b4ea9dbb0c52e8c392f1).

Lack of knowledge and awareness of headache disorders needs to be addressed at all levels of society, to dismantle the barriers to achieving positive brain health outcomes. Much has been done by the Global Campaign against Headache, including the publication of around 60 population-based scientific articles addressing headache-attributed burden [2] and proposals, with economic evaluation, for the organization of headache services [14–16]. The joint work of scientific and patients’ association has increased global awareness, but still this has not been enough.

#### 1.1 Advocacy

Public and political awareness of the burden and impacts of headache disorders are of crucial importance in the promotion of better headache care and headache prevention as well as on stigma reduction [20, 21]. Advocacy, directly involving people with headache, represents the first step in raising awareness and towards better understanding of headache disorders as well as in reducing stigma and discrimination, especially in the workplace.

#### 1.2 Policy, plans and legislation

Collaboration between associations of people with headache disorders, clinicians, researchers, industry and policymakers is essential to facilitate the development and implementation of evidence-based policies and plans across sectors, in particular for the organization of health services and for the labour sector. As it is stated in the Global Campaign’s background: “Headache disorders do not shorten life. This is one reason why they are so poorly acknowledged. On the other hand, they impose pain and personal suffering, which may be substantial, damage quality of life and cause financial losses. Above all, headache disorders are disabling and often have comorbidities.”

It is of importance that an adequate recognition of the comorbidity and multimorbidity profile associated with headaches disorders [22] is considered, since numerous opportunities exist to integrate care pathways and therapies for people with headache disorders and associated conditions, such as mental health and cardiovascular diseases. The likelihood that patients with headache disorders are also patients with other disorders, calls not only for strengthening the efforts of associations and organizations of people with headache disorders but also for fostering their collaboration with other organizations as partners in the implementation of policies for headache disorders. Attention should be placed in reviewing disability and other relevant policies and laws to be more inclusive of people with headache disorders, including by reviewing criteria to access disability benefits; providing funding to support people with headache in employment; making working environments more accessible with employment regulations and labour laws that govern the public and private sectors so as to make environment a facilitator for headache sufferers.

#### 1.3 Financing

Headache disorders, both in HICs and in LMICs, represent burdensome conditions not only for individuals but also for societies in part because of the costs associated with diagnosis and management; but far more because of the productivity losses attributed to these highly prevalent disorders among adults of working age (these indirect costs may account for more than 90% of total headache costs [23]). Much of these costs could be remedied by prevention, early detection and timely treatment: in fact, different approaches to treatment showed that effectiveness on disease severity and frequency of attacks is also accompanied by cost-effectiveness (see the following studies referred to the last few years, not intended to be comprehensive [24–31]).

Appropriately funded policies and investment in the implementation of healthcare programmes required to ensure access for all people with headache disorders to diagnosis, treatment and care, should therefore, not only for people with headache disorders to reduce the financial impact of out-of-pocket healthcare costs but also be offset potentially to a very large extent by a reduction in the indirect costs of headache disorders [23]. However, the studies on economic evaluation are often conducted following the occidental economic model, without considering the needs and the context in Asia or in LMIC countries. Left unaddressed are the real barriers of access to treatment in the poorest countries in the world. The financial burden of headache disorders needs to be a public health priority, especially for LMICs where costs of medications are considered the leading barrier to access to new available treatments. Furthermore, in these counties there are scarce consideration about economic evaluation studies, rather than HICs. It can be due to less developed reimbursement systems and to an absence of officially established willingness-to-pay threshold that helps to define which treatments are considered cost-effectiveness [32]. Such a problem in part persists also in HICs, as poor information on the economic evaluations among underserved populations in wealthier countries are lacking. In wealthier regions, access to specialized headache clinics, neurologists, and advanced treatment options might be more readily available, resulting in better care for those suffering from migraines or chronic headaches. Disparities persist within these countries, affecting marginalized communities or remote areas where healthcare resources are limited. Factors like socioeconomic status, geographic location, and cultural barriers often create hurdles in accessing specialized care and adequate treatment for headache disorders, exacerbating the divide in healthcare outcomes [33, 34]. Efforts to address these disparities involve a multifaceted approach, including community outreach programs, increased education, improved healthcare infrastructure in underserved areas, and policies aimed at ensuring equitable distribution of resources for headache disorder management [17].

Patients’ associations should be directly involved in the process of fund allocation by providing inputs on the way in which funding should adequately represent and include also rare headaches, or incorporate services and treatments that are unequally available worldwide.

### Strategic objective 2: providing effective, timely and responsive diagnosis, treatment and care

Headache disorders are leading causes of morbidity and disability, and are moreover associated to a large amount of comorbidities [20]. They are ubiquitous; therefore, their mitigation requires equitable access for all effective health care services, and to labour sector services aimed at promoting working environments that are not hostile to those with headache [4].

Unfortunately, diagnostic delay or misdiagnosis are often experienced for the rarest forms of headache, such as cluster headache or other trigeminal autonomic cephalalgias [35–38], but also in migraine [39–43], with clear effects on increased disability and disease burden.

#### 2.1 Care pathways

Services and care pathways should include access to quality emergency care and be responsive to the needs of people with headache disorders, including their caregivers and family members. Such pathways should be distributed so as to offer equal ensure all, whether living in either urban or rural areas. They should be tailored to the needs of all age groups and oriented to each stage of the life course, from pregnancy through early childhood to care for older adults, with special consideration of the transition from adolescence to adulthood [44, 45], meeting the needs of paediatric populations [46–49] and special groups such as pregnant women. They should be inclusive of vulnerable population groups, including socioeconomically disadvantaged as well as refugees, displaced populations and migrants.

For all these reasons, headache care should be based in and integrated with primary health-care services, and organized to provide access to secondary and tertiary health care levels when and only when clinically appropriate. Such a pathway is necessary to offer equitable access to large numbers of people, while promoting adequate and timely diagnosis and prescription of best available therapies for acute and preventive management, tailored according to the clinical needs of each patient [14]. In all environments, digital health solutions are increasingly an option, with some evidence of effectiveness [50], enabling remote tele-health consultations [51–55]. As diagnosis is a key to successful treatment, and as migraine, among other headache disorders is underdiagnosed, it should be noted that migraine meets most but not all criteria for a policy recommendation for screening [56, 57]. This is an area for future exploration, and it is particularly important in LMIC. In fact, headaches constitute the most common patient reasons for primary care encounter in LMICs [58], whereas it ranked only at the sixth position in HICs [59]. Secondary headaches in particular constitute a matter of concern due to the limited availability of instruments for differential diagnosis. A recent cross-sectional investigation showed that approximately one-third of headaches were found to be secondary in African, Middle-East and Asian countries, the most common subtype of secondary headaches was headache attributed to substances or their withdrawal [60]. However, several primary causes exist that need to be ruled out with laboratory (e.g. infections) and neuroimaging investigations (e.g. brain tumors) [61] which are less available in LMICs.

#### 2.2 Medicines, diagnostics and other health products

The management and treatment of headache disorders is mostly pharmacological, although other therapeutic approaches include behavioural, invasive and non-invasive neuromodulation, and nutraceuticals [8, 13, 62–66]. In all cases management should follow local guidelines that take due account of resources, but which guarantee access to all to appropriate care on an equitable basis, and to drugs on national essential medicines lists when needed.

Advanced diagnostic procedures, including neuroimaging, are rarely needed, and their use incurs not only on financial cost but also, in resource-limited settings, opportunity costs (depriving other patients who might need them). They should be reserved for the exclusion of secondary headaches [61].

#### 2.3 Health workers’ capacity-building, training and support

Since 2004 the WHO Atlas Country resources for neurological services, revised in 2017, highlighted the lack of neurological services and of personnel to take care of the increasing number of neurological patients (see: https://www.who.int/publications/i/item/atlas-country-resources-for-neurological-disorders/). The global current and projected neurology mismatch in supply and demand has harmful consequences: reduced and delayed access to high-quality care, worsened patient outcomes, and eroded career satisfaction for neurologists. The gap between demand for and supply of neurological services is widening as a result of several factors, including population ageing and growth, which will further increase the number of people who develop neurological disorders. For this reason, for people with headache disorders, improved health outcomes will greatly depend on enhancing skills in primary care, whether this is provided by general practitioners (GPs), clinical officers, pharmacists or nurses. Of course, an adequate neurological workforce is also a necessary aim, including both adult and child neurologists, as are the availability of other specialist health care providers, in particular radiologists, paediatricians, gynaecologists, psychiatrists and psychologists.

The training and education of an interdisciplinary workforce, is required to support the delivery of person-centred care to people with headache disorders and improve their overall health and quality of life. Increasing the whole level of knowledge on headache disorders among clinicians is of core importance to pursue the objective of providing different levels of care. If headache treatments should start from primary care providers and pharmacies, then people who work in such contexts need to acquire the knowledge and skills to propose over-the-counter medications and suggest when to address second-level care. Some experiences on training and education issues are reported in literature [67–73]. Taken as a whole, these experiences mostly show that academic medical education on headaches is not satisfactory in terms of the amount of hours of attendance to headache clinics, and of the amount of medical students or residents who learned how to take headache history: thus, future clinicians will surely benefit from an increase of education at different levels, from undergraduate training to specialist residency.

Medical students (who are the future GPs and specialists), along with current specialists in adult and child neurology, should of course be the primary recipients of enhanced headache training. However, considering the urgent need to develop skills in managing headache disorders in primary care, training and support must, as a priority, be given to GPs and pharmacists, and to clinical officers and nurses working in primary care. For multidisciplinary treatment, specific training should also be addressed to specialists in radiology, paediatrics, gynaecology, psychiatry and psychology, who may be involved at different levels in the management of patients with headaches. Despite efforts of many regional and international societies to educate and train a new generation of headache experts (e.g. the European Headache Federation School of Advanced Studies which held 15 high-level training events, see https://www.ehf-headache.com/news-events/sas), still the gap remains unfilled and the shortage of headache specialist impacts on quality of care worldwide.

#### 2.4 Carer support

Family burden is a relevant, but often poorly recognized, part of the headache burden, which may take different forms according to the person with headache and their carer [4, 74–82], and include: adult caregiving for adult, adult for child or adolescent, and the more complex situation of child or adolescents dealing with a parent suffering from headache.

The challenges caregivers’ faces include stress, role strain, financial burden, and social isolation, depending on the ages of both the caregiver and the person with headache, and may affect caregivers’ own well-being and social relationships. Information to caregivers on how to deal with a person with headache disorders should be part of the clinical path, at least in the format of information and educational materials. The role of national and international patients’ associations is crucial to reach this objectives and further work needs to be done in all those countries where patients’ associations do not exist.

### Strategic objective 3: implementing strategies for health promotion and headaches prevention

Headache disorders arise out of the interaction between genetic predisposition and environmental or lifestyle risk factors. Among the latter, some are not modifiable (e.g. female sex for migraine) but others may be modifiable (e.g. poor sleep, poor diet, physical inactivity, medication overuse, stress, dehydration), offering room for prevention.

Behavioural risk factors and risk factors that can be addressed though targeting behaviours are modifiable with a life course approach. Most of them have been especially reported for the development of chronic migraine and include: overuse of acute migraine medication, ineffective acute treatment, obesity, diabetes, hypertension, stressful life events, night-shifts on work, depression and other psychiatric comorbidities, sleep problems, temporomandibular disorders, physical inactivity, smoking, alcohol consumption [20, 83–90]. In turn, headache disorders, and migraine in particular, are risk factors for other conditions, among all cerebrovascular ones [91–96], but also other cardiovascular, visual, perceptual and psychiatric disorders [97–99].

#### 3.1 Promoting healthy behaviour across the life course

The literature shows that many headache disorders might be ameliorated by modifying risk factors, as has been the case for many other non-communicable diseases, for which primary and secondary prevention campaigns exist. The degree to which headache can be prevented, to the best of our knowledge, has not been systematically addressed in population studies, and no indication on risk factors for headache-related burden are available in GBD estimates, one of the main reasons being the overall lack of “headache” in the main large population studies, as shown in some recent studies who relied on an aggregate dataset from different population surveys [100–102]. Nevertheless, evidence on the occurrence of headaches, and migraine in particular, in association with several risk factors exists, and some of these risk factors (e.g. sleep hygiene, adequate hydration, moderate physical activity, control over medications intake) are part of the indication given to patients as part of patients’ education, in particular for the treatment of chronic headaches [103, 104].

Addressing the main risk factors through health promotion and diseases prevention, should therefore be part of the routine clinical activity of headache specialists, but clearly this is not enough: a life-long process of education on the reduction of modifiable risk factors should be regularly carried out, beginning from childhood, and in particular among those with known family association with migraine. Such a primary prevention activity is of great importance, not only for the reduction of risk of developing headache disorders, but also to impact on avoiding those severe conditions for whom headaches represent a risk factors, such as cerebrovascular conditions. As stated in the WHoiGAP also for headache is true that “Universal Health Coverage represents a key component for promoting brain health and well-being. An important element includes addressing social and economic determinants through a coordinated intersectoral response in a gender-sensitive manner. Collaboration with local populations, including indigenous people, should be undertaken to explore culturally appropriate ways of preventing neurological disorders that respect local customs and values” [1].

#### 3.2 Infectious disease control

Secondary headaches caused by infections are deemed to be rare, at least in high income countries or regions, the main reasons lying in the reduced prevalence of infective disorders commonly associated headaches, in particular meningitis and encephalitis [105–108].

However, COVID-19 pandemic determined an increased attention on headaches as one of the possible consequences of a systemic infection. A recent meta-analysis based on 35 studies and 28,438 patients addressed headache prevalence during acute phase of COVID-19 and up to six months [109]. In the acute phase, headache was shown in 47.1% (95% CI: 35.8–58.6%) of the patients at symptoms’ onset/hospital admission (31.1% among hospitalized and 58% among non-hospitalized patients), whereas prevalence was lower during the post-COVID phase, i.e. 10.2% at 30 days, 16.5% at 60 days, 10.6% at 90 days, and 8.4% after 180 days since onset/hospital discharge. In addition to this, headache emerged as the third most common symptom after vaccination against SARS-CoV-2: in a meta-analysis of 84 studies and 1.57 million participants [110], it was detected in 22% (95% CI 18–27%) of subjects after the first dose of vaccine and in 29% (95% CI 23–35%) after the second (compared to 10–12% of placebo recipients), and mostly remit within 24 h.

Although pandemic has been declared over in March 2023 by the WHO, it is still too early to understand and clearly define whether long-term effects of COVID-19, will determine in some patients a chronic course of a secondary headaches. Likely, there will be patients who will develop a stable pattern, whereas others will experience a remission of headache. To date treatment for long-COVID headache disorders do not differ from care of headaches in general, no specific pathway and plan is available for the treatment of such condition [111, 112], which therefore needs to be adequately recognized in a frame of collaboration between experts, stakeholders and associations from different fields.

#### 3.3 Preventing head/spinal trauma and associated disabilities

Post-traumatic headaches (PTH) is a secondary headaches following traumatic brain injury (TBI) usually developing within 7 days from the injury [61]. It may be very common, especially in the first months: a study identified PTH in 27–33% of patients over a six-month period after mild TBI [113]. It may progress to chronic and sometimes debilitating conditions, defined persistent PTH, if it does not resolve within 3 months. Few epidemiological data on PTH exist: the 1-year prevalence of persistent PTH has been estimated at 0.21%, and lifetime prevalence at of 2.4–4.7% [114, 115], but the data are uncertain because mild TBI, although apparently a cause of PTH, is often unrecognised. The mechanisms that lead some patients, and others not, to develop PTH are still unclear [116].

To limit PTH and its long-terms effects, actions should target the primary cause. To a large extent this means improving sport safety and road safety, alcohol or drug consumption, non-use of helmets, lack of seat belts and child restraints and inadequate enforcement of traffic laws. The interventions – especially educational ones – should also address unsafe home and community environments, as well as increase responsiveness in post-injury, such as neurosurgery and neurorehabilitation where available, and long-term care for those who suffered from TBI.

#### 3.4 Reducing environmental risks

Smoking is often reported as a trigger for migraine headache and, as shown in a review, headache and tobacco exposure remain associated despite the presence of several biases in studies [117]. Tobacco exposure also seems to be associated with a worse clinical profile, i.e. chronification, in cluster headache [118]. More in general, headache and migraine in particular, seem to be associated to a set of environmental triggers, including barometric pressure change, bright light, air quality, presence of smell and environmental noise [119, 120].

Such triggers often exert their negative effect on work-related tasks and actions are needed to mitigate such triggers and create a migraine-friendly workplace. A recent initiative was launched by the European Headache and Migraine Alliance (EMHA) with the Migraine Friendly Workplace Stamp, an initiative aimed to raise awareness and respect towards people with migraine in the workplace. Benefits of such an initiative are different and include, among the others, increased productivity, creation of long-term employee loyalty, improved organization reputation. Evidence exists of the huge socio-economic burden of migraine in the workplace [22, 121, 122]. Migraine education programs in the workplace, employer sponsored migraine education and management/referral programs, migraine-friendly work environments, headache care access and treatment optimization are strategies that need to be implemented to reduce the overall burden of headache by acting on the environmental risk factors that act on the most burdensome headache disorder and in the context in which it mostly produces its effects [123].

#### 3.5 Promotion of optimal brain development in children and adolescents

Promoting optimal brain development in childhood and adolescence which, among the others, aim at reducing the burden of headaches disorders requires action on different aspects. In particular, literature shows an association between the development and maintenance of headache disorders and negative childhood early life events, including childhood maltreatment [124–127], low socioeconomic status and education [128–130] and associated conditions such as sleep disorders and obesity [131–135].

Part of these aspects can be prevented, or at least their effect be remedied during adolescence and transition to adulthood. The most relevant predictor of adult headache is, however, paediatric headache which is likely to continue in adulthood if untreated. This information can be retrieved in anamnesis in clinical practice, but is quite difficult to get from population studies. It is estimated that around 60% of children and adolescents suffer from headache and around 8% from migraine over periods varying from 3 months to lifetime [136]. This study also found that migraine prevalence is 5.8% among subjects below the age of 14, whereas it is 7.7% considering the whole set of subjects aged below 20 [136]: such figures then reach 14% in adulthood.

Although longitudinal analyses are not systematically reported, the rising trend in prevalence suggests that early intervention, either pharmacological or non-pharmacological, is of importance in reducing the overall burden of headache disorders. Focussing on prevention of children and adolescents’ headache disorders by eliminating risk factors and promoting health behaviours, could lead to strengthen national capacity for the promotion of optimal brain development in children and adolescents in general.

### Strategic objective 4: fostering research and innovation and strengthen information systems

Good evidence is the key to adequately informed health policy. In the last years, the amount of research in the field of headache disorders has been rising due to several reasons and mostly to the increasing number of different trials on new drugs such as monoclonal antibodies [137, 138], as well as due to the studies that are associated to them, e.g. real-life studies, studies on their economic assessment or updates on guidelines. Also, research on SARS-CoV-2 or long COVID-19 and headaches was not irrelevant: in fact, for example, only between January 2020 and June 2022, a total of 2,100 articles addressing headaches and COVID-19 in title or abstract, out of 15,500 have bene published. In other words, a sixth of published research on headache disorders dealt with COVID-19 infection.

However, several areas exist that might benefit from research and innovation in the next decade, and from an international approach that fosters research collaborations, including data-sharing, in order to reduce duplication, identify knowledge gaps, fast-track innovation and build capacity in low-income settings. As stated in WHOiGAP and really true for headache research “better representation of low- and middle income countries in the neuroscience research environment should also acknowledge country-specific and local needs so that strategies for diagnosis and management of neurological disorders are tailored to the context” [1].

In addition, the active participation of patients and or of their organizations is crucial: there is no research without patients’ participation, which should not only be included when the trial is ongoing as experimental subjects, but also as subjects actively involved in the process of outcome definition, thus from planning to implementation.

#### 4.1 Investment in research

Given the non-decreasing trends in headache disorders epidemiology and related global disability and burden, research is particularly needed in those areas in which knowledge is limited, but also in the areas of service provision and health systems organization and care implementation. The reason behind this is that headache disorders are extremely prevalent and cause a substantial burden to individuals and societies. Despite headache is one of a leading cause of disability, the research funding allocation in this field continued to remain very low worldwide.

Basic research in the latest years brought to the development of new compounds for migraine prophylaxis, and also to the development of non-invasive neuromodulation devices. However, the efficacy that is found in RCT is usually different from the effectiveness found in large real-life studies. In turn, such effectiveness might not correspond to a similar impact on societal burden, the reason for this being the difficulty in the process of delivering of the most appropriate treatment to each patient, i.e. achieving personalized medicine and the persistence of societal barriers and policy, services, systems levels [139].

So, research in the field of headache disorders should focus not only on RCTs, but also on pragmatic studies, which enable comparing different kinds of treatments which share the same clinical indication, and that enrol participants that are similar to patients who would receive the intervention if it became usual care [140]. Furthermore, large scale population studies should make an explicit mention to headache disorders as source of pain. Two studies based on a harmonized dataset derived from different studies [102], showed that pain prevalence over 10 years is expected to increase by 10–20%, and that the onset of pain, in subjects previously not referring pain, was predicted by modifiable factors such as fatigue sleep disorders and obesity. As the primary source of pain was not specified, both studies [100, 101] had to interpret data on pain prevalence hypothesizing that the main reason for pain development was either headache or back pain, which is reasonable, in consideration of the age group to which most participants belong. More population-based studies on headache are needed, especially in the LMICs, due to the poor availability of information about headache in low-resource settings, despite the existent evidences suggest that headache disorders occur on a similar scale in LMICs as they do in HICs [33, 141].

Public health research on headaches should consider how many inequities in health care and the lack of access to care for many populations of LMIC impacts on the burden of headache disorders. This type of research should also show if and how countries are far from the goal of Universal Health Coverage (UHC) for all, a goal also supported by the UN SDG 2023 campaign. Achieving UHC means granting all people access to the full range of quality health services they need, when and where they need them, without financial hardship, and it involves the full continuum of essential health services: health promotion, prevention, treatment, rehabilitation and palliative care [142]. Every country has a different path to achieving UHC and deciding what to cover based on the needs of their people and the resources at hand. However, the importance of access to health services and information as a basic human right is universal. Public health research should focus on how lack of UHC impacts on these “invisible diseases”.

The different countries, through their relevant private and public agencies, should therefore increase the amount of investment in research on headache disorders, keeping in mind the need to allocate resources on the different areas. This means supporting independent research on headache disorders, addressing the main different clinical conditions (which is of outmost importance since the vast majority of headache research is on migraine), and addressing different topics: all of them, e.g. basic research, RCTs, studies on treatment delivery, population studies, public health and pragmatic trials, should receive adequate investments.

#### 4.2 Data and information systems

Data are the essential foundation of informed policy, but data collection is both time and resource consuming. Data sharing is a key to reduce the costs of research, that might be incurred through duplication, while stimulating innovation and encouraging collaboration not only in responding to research questions but also in the formulation and evaluation of policy. The International Headache Society has only recently released its first guidelines on headache registries [141], addressing the need of core elements. These should include validated headache-specific questionnaires, patient reported outcome measures, and medical record data, whereas other elements (e.g. pharmacy claims data, biospecimens, and neuroimaging data) might be included based on the aims of the registry. Several registries are currently available and in the last few years many publications have been made based on the data collected in these registries [143–156]: however, such an effort was partly thwarted by the inconsistency between the main elements in these registries, which will make it necessary to undertake harmonization procedures which will reduce the richness and complexity of the original information.

Associations of people with headache disorder must be involved in the process of registries creation and at the same time, the associations should work towards raising awareness on the importance of registries: the right to count for societies must pass from the engagement in being counted, in order to fight discrimination and promote equal access to treatment for people with headache disorders.

## The headache revolution: from headache burden to a global action plan to implement and strengthen the public health approach to headache disorders

Like brain health, that affects all of us, also headaches, that affect millions, are invisible. The objectives of WHOiGAP are ambitious and require strong commitment from WHO Member States and global organisations if they are to be achieved over the next decade. Reducing neurological burden improves global brain health defined by WHO as “the state of brain functioning across cognitive, sensory, social-emotional, behavioural and motor domains, allowing a person to realize their full potential over the life course, irrespective of the presence or absence of disorders” [157]. With 75% of the burden of nervous system disorders falling on LMICs, global collaboration is essential. Several global and regional neurological scientific and lay associations have set out defined their plans for implementation of WHOiGAP, including but not limited to the World Federation of Neurology, International Bureau for Epilepsy, International League Against Epilepsy, International Child Neurology Association and European Academy of Neurology. The WHOiGAP will be regularly reviewed by member states, and interim reports will be prepared for the World Health Assembly, in 2025, 2028 and 2031 [158, 159] and these dates set targets for the steps to be achieved by all stakeholders.

Successful implementation of WHOiGAP will represent a revolution in neurology, not only by increasing public awareness of brain disorders but also by boosting numbers of neurologists in training, increasing research funding, and making neurology a priority for policy makers. The community of clinicians and researchers that work in the field of headache disorders must be part of this revolution. They have to make the invisible disorders visible into the public health scenarios. Clearly, there are major challenges to overcome in addressing the burden of headache disorders, but, for both scientific and lay headaches societies around the globe, the chance to act is here and now: achieving this aim means improving the health and wellbeing of people with headache and thereby contributing to increased brain health and increased global health.

The headache revolution has already begun and must go on.

## Data Availability

Not applicable.
